# A Multi-Agent Reinforcement Learning Method for Omnidirectional Walking of Bipedal Robots

**DOI:** 10.3390/biomimetics8080616

**Published:** 2023-12-16

**Authors:** Haiming Mou, Jie Xue, Jian Liu, Zhen Feng, Qingdu Li, Jianwei Zhang

**Affiliations:** 1 School of Optoelectronic Information and Computer Engineering, University of Shanghai for Science and Technology, Shanghai 200093, China; 201440059@st.usst.edu.cn (H.M.); 221240078@st.usst.edu.cn (J.X.); 213330674@st.usst.edu.cn (Z.F.); 2 Institute of Machine Intelligence, University of Shanghai for Science and Technology, Shanghai 200093, China; liujianwgx@163.com; 3 Department of Informatics, University of Hamburg, 20146 Hamburg, Germany; jianwei.zhang@uni-hamburg.de

**Keywords:** omnidirectional walking, bipedal robot, multi-agent reinforcement learning, experience replay mechanism, curriculum learning

## Abstract

Achieving omnidirectional walking for bipedal robots is considered one of the most challenging tasks in robotics technology. Reinforcement learning (RL) methods have proved effective in bipedal walking tasks. However, most existing methods use state machines to switch between multiple policies and achieve omnidirectional gait, which results in shaking during the policy switching process for bipedal robots. To achieve a seamless transition between omnidirectional gait and transient motion for full-size bipedal robots, we propose a novel multi-agent RL method. Firstly, a multi-agent RL algorithm based on the actor–critic framework is designed, and policy entropy is introduced to improve exploration efficiency. By learning agents with parallel initial state distributions, we minimize reliance on gait planner effectiveness in the Robot Operating System (ROS). Additionally, we design a novel heterogeneous policy experience replay mechanism based on Euclidean distance. Secondly, considering the periodicity of bipedal robot walking, we develop a new periodic gait function. Including periodic objectives in the policy can accelerate the convergence speed of training periodic gait functions. Finally, to enhance the robustness of the policy, we construct a novel curriculum learning method by discretizing Gaussian distribution and incorporate it into the robot’s training task. Our method is validated in a simulation environment, and the results show that our method can achieve multiple gaits through a policy network and achieve smooth transitions between different gaits.

## 1. Introduction

As fundamental research in robotics continually advances, service robots are increasingly permeating our daily lives [1]. In specific application scenarios, bipedal robots demonstrate greater flexibility and efficiency than their wheeled counterparts, making them a significant subject within the field of robotics research. Gait optimization is foundational to the normal operation of bipedal robots, chiefly referring to the robots’ ability to achieve rapid, stable locomotion through self-balancing [2,3]. To enable balanced motion in bipedal robots, gait optimization needs to circumvent leg–foot collisions during robot movement. Traditional gait control methods, such as human walking parameters [4,5], zero moment point (ZMP) [6,7], passive walking [8], fuzzy logic control [9], and optimization algorithms [10,11], have been consistently employed in bipedal robot gait control for many years. However, these traditional methods present problems. Specifically, human walking parameter methods require time-intensive manual parameter tuning, often resulting in less than optimal values. ZMP methods, on the other hand, present several drawbacks, such as insufficient energy, restricted walking speed, and limited resistance to external disturbances [6]. These traditional techniques exhibit high computational complexity, low robustness, and poor generality. While Model Predictive Control (MPC) is not classified as a traditional gait control method, its wide application in bipedal robot gait control is noticeable [12]. Reference [13] proposes a holistic MPC scheme based on differential dynamic programming to tackle the challenges of physical constraints and model discrepancies in bipedal robots.

RL methods are widely utilized for bipedal robot control due to their aptitude for model-free learning [14,15]. RL techniques offer benefits such as reduced hardware requirements for robots and significant time savings in the debugging process [16]. However, choosing an appropriate learning rate for RL-based methods poses a practical challenge. A small learning rate may result in protracted training progress, whereas a large one could trigger oscillations or even algorithm divergence, compromising training performance [17]. Integrating RL with neural networks has provided some solutions for this issue [18,19]. The robot interacts with the environment and learns through continuous trial and error to obtain a reward function to judge whether the skill is good or bad, and eventually learns the skill. This learning approach circumvents inaccuracies induced by mathematical models and bolsters the robustness of the training process [20]. The Proximal Policy Optimization (PPO) algorithm, a popular actor–critic algorithm, is frequently used for training bipedal walking [21,22]. It facilitates direct control through end-to-end learning, broadening the applicability of RL. PPO-based model-free learning for bipedal walking gravitates around multi-action policy learning algorithms based on Markov Decision Processes (MDPs) [18,23]. Presently, the PPO algorithm has emerged as the standard method for training bipedal robot gaits. However, it faces challenges concerning slow training speed and low sample utilization in gait optimization. To resolve these issues, an experience replay mechanism has been introduced, which increases past data replay frequency and curbs resource wastage, thereby enhancing the convergence speed and efficiency of RL algorithms [24]. Nonetheless, the experience replay mechanism also has its limitations, including high storage space requirements, substantial computational load, and demanding hardware prerequisites. Consequently, employing a heterogeneous strategy can mitigate the computational overhead associated with RL algorithms. Although leveraging heterogeneous RL algorithms significantly accelerates the training process through multi-threading techniques, a considerable amount of training time is still required [25]. Moreover, existing off-policy RL algorithms do not fully take advantage of good and general experiences since they depend on replaying individual experiences. Model-based RL methods can improve exploration efficiency by leveraging partial knowledge of the environment, thereby minimizing ineffective exploration. The amalgamation of MPC and RL can effectively carry out safe exploration [26]. However, these methods might not entirely utilize the robot’s capabilities, potentially resulting in suboptimal policies.

Unfortunately, the majority of current gait research tends to focus predominantly on straight-line gaits or turning gaits. Turning gaits are often achieved by integrating the PPO algorithm with curriculum learning methods. Existing research suggests that training strategies incorporating curriculum learning can present advantages in generating complex actions. A common approach for training bipedal robots for omnidirectional walking is to commence with straight-line movement training, and then progressively introduce larger turning angles. This gradual progression method has been demonstrated to effectively bolster the training success rate of complex strategies. However, curriculum learning methods are prone to catastrophic forgetting, wherein the difficulty of early training stages is lost, posing challenges in achieving multiple gait patterns using a single policy network. Unlike previously mentioned RL gait control methods that require switching between multiple policy networks, our approach allows for omnidirectional gait using a single policy network. This mitigates the constraints of controller switching and facilitates more fluid transitions between different gaits. Specifically, this paper offers the following advancements:

(1) A novel approach to multi-agent RL is constructed which is based on the actor–critic framework. A combinatorial optimization approach is employed to maximize the cumulative reward and policy entropy. Additionally, a new experience replay mechanism is designed specifically for multi-agent RL methods.
(2) A new periodic gait function is designed and incorporated as an objective into our RL policy. Our periodic gait function enables bipedal gaits to exhibit symmetry and periodicity.
(3) A curriculum learning method based on Gaussian distribution discretization is proposed. During the training process, the turning angle is dynamically adjusted, and a single strategy is used to achieve omnidirectional walking with different turning angles.

## 2. Related Work

Traditional gait optimization typically involves planning joint trajectories based on walking requirements, followed by the calculation of joint angles using an inverse kinematic model. The goal is to ensure stability, avert falls, and enhance aesthetic appeal. To tackle the complexities associated with multi-link structured bipedal robots, researchers have conceived simplified models, such as the three-mass inverted pendulum model. This model takes into account the influence of the support leg on motion stability, reducing the robot to three crucial components: the center of mass (COM) of the torso, the COM of the support leg, and the COM of the swing leg during the single-leg support phase [27]. This streamlined framework aids in studying motion stability and control strategies in bipedal locomotion.

In [28], a contemporary online optimization technique is presented, enabling humanoid robots to execute vertical jumps effectively by controlling the centroidal angular momentum and mitigating the impact upon landing. Nevertheless, manual parameter adjustment is not only time-consuming but also does not guarantee the generation of optimal values. Consequently, numerous scholars have suggested optimization algorithms for robot training. At present, bio-inspired optimization algorithms are extensively applied to bipedal robot gait optimization. However, owing to the abundance of robot parameters, bio-inspired optimization algorithms are prone to becoming ensnared in local optimization during the optimization process, thus making it challenging to achieve the robot’s maximum walking speed.

RL methods have demonstrated significant potential and the capacity to supplant traditional model-based controllers in various studies. Remarkably, significant progress has been achieved using RL methods for tasks such as walking and jumping on the Cassie robot [29,30]. In a study of Cassie walking gaits by Jonah Siekmann et al. [31], a clock signal-based reward function was employed to attain periodic walking gaits with swing and stance phases for each leg. The study in reference [32] utilizes domain randomization techniques to facilitate policy learning adaptation to variations in system dynamics, thereby achieving robust behaviors. In [33], a formulation to counteract the limitations of RL controllers for bipedal robots is proposed by integrating footstep constraints. This formulation allows the learned controllers to maintain robust and dynamic gaits while adhering to external environmental constraints. The study in reference [23] leverages model-free RL techniques to fine-tune the base policy, adapting it to an imperfect extrinsics estimator, and demonstrates successful transference to a physical robot. RL has also been used to learn diverse motion skills and achieve seamless transitions between them. Yu et al. utilize precomputed trajectory data and terminal rewards to learn specific turning gaits for bipedal robots, enabling seamless transitions in the turning process [34]. In reference [35], a policy learning method based on footprint planning is designed to accomplish omnidirectional walking on 2D and 3D terrains by following a learning strategy for a given sequence of steps in the ROS framework. Rodriguez et al. propose a novel approach for achieving omnidirectional gaits for bipedal robots [36]. They amalgamate RL with a curriculum learning approach, progressively increasing the difficulty of gait training tasks. This integration enables the learning of robust and versatile gaits in various directions.

## 3. Preliminaries

### 3.1. Actor–Critic Algorithm

Central to the concept of RL is an intelligent agent engaging with an environment while executing a specific task. This interaction involves taking actions and receiving reward signals from the environment, which indicate the success or failure of the agent’s actions. As the agent continually interacts with the environment, it refines its action selection strategy based on the received feedback, aiming to maximize cumulative rewards. Through numerous iterations, the agent eventually unearths the optimal strategy to complete the assigned task. During the actual interaction between the agent and the environment, constraining the number of interactions could result in a significant discrepancy between the presumed reward sequence and the actual values, thereby exacerbating the variance of the reward signals. Moreover, traditional policy gradient algorithms often exhibit a protracted convergence speed. One possible resolution to these issues is the utilization of an actor–critic algorithm, merging the roles of an actor and a critic and leveraging joint training to bolster stability and learning efficiency. In the actor–critic algorithm, the actor is tasked with action selection, while the critic evaluates the value of these actions. The actor generates actions $a_{t}$ based on the current state and policy $\pi (a_{t}|s_{t},\theta )$, where θ represents the parameters of the policy network. The critic estimates the action value function $Q(s_{t},a_{t},\theta_{q})$ or the advantage function $A(s_{t},a_{t},\theta_{a})$ based on the current state and action, where $\theta_{q}$ and $\theta_{a}$ represent the parameters of the critic network. The goal of the actor is to maximize the total return $J\left(\theta \right)=E_{\pi }\left[R\right]$, where *R* represents the return value. To achieve this goal, the actor utilizes policy gradient methods to update the parameters θ by maximizing the expected value of the policy value function $V(s_{t},\theta )$:

(1) $$\nabla_{\theta }J\left(\theta \right)\approx \frac{1}{N}\sum_{t=1}^{N}\nabla_{\theta }\log\pi \left(a_{t}|s_{t},\theta \right)A\left(s_{t},a_{t},\theta_{a}\right),$$

where *N* denotes the number of samples. By iteratively updating the parameters θ, the actor optimizes the policy to choose actions that yield higher returns.

The critic estimates the action value function or advantage function based on the chosen actions and the true returns. Specifically, the critic aims to minimize the difference between the action value function or advantage function and the true return using the following loss functions:

(2) $$L\left(\theta_{q}\right)=\frac{1}{2}E_{\pi }\left[{\left(Q\left(s_{t},a_{t},\theta_{q}\right)-R_{t}\right)}^{2}\right],$$

(3) $$L\left(\theta_{a}\right)=\frac{1}{2}E_{\pi }\left[{\left(A\left(s_{t},a_{t},\theta_{a}\right)-R_{t}\right)}^{2}\right],$$

where $R_{t}$ represents the true return. Through gradient descent, the parameters $\theta_{q}$ and $\theta_{a}$ of the critic network are updated to improve the accuracy of the estimation for the action value function or advantage function.

By training and updating the actor and critic together, the actor–critic algorithm can learn more stably and find better policies in complex environments. The key feature of this algorithm is the simultaneous training of both the policy and the value function through the estimation of action values.

### 3.2. Robotics Platform

To conduct bipedal locomotion studies, we construct our bipedal robot in a simulated environment. The robot stands at a height of 133 cm and weighs 30 kg. It comprises a total of 20 degrees of freedom, evenly split with 10 degrees of freedom allocated to the upper body and an equivalent number to the lower body. To focus on the qualitative aspects of the walking gait, the 10 degrees of freedom corresponding to the upper body, arms, and hands are immobilized, activating only the hip joints and the remaining 10 degrees of freedom related to the legs. The joint parameters are outlined in Table 1.

**Figure 1:**
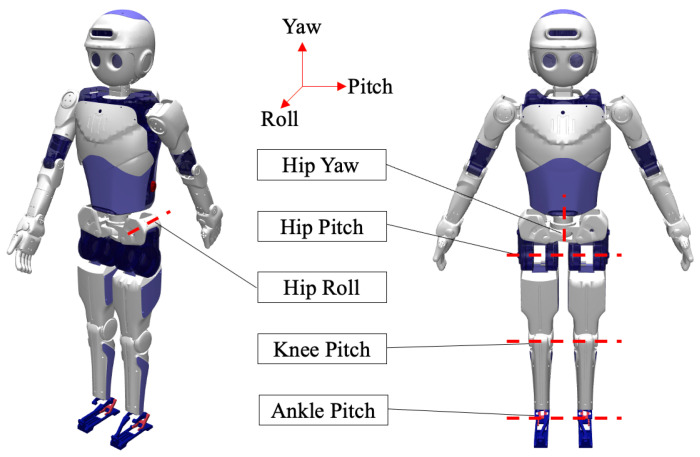
Appearance and structure diagram of humanoid robot. In the simulation, we locked the joints of the upper body and only had 10 joints belonging to the lower body.

Figure 1 presents the distribution of the lower body joints and actuators, encompassing hip yaw, hip roll, hip pitch, knee pitch, and ankle pitch. To monitor the robot’s body posture, an inertial measurement unit (IMU), the 3DM-GX5-25, is installed 5 cm above the hip yaw degree of freedom. This IMU allows for the measurement of the robot’s center of mass roll, pitch, and yaw angles, along with the estimation of the center of mass’s horizontal velocity, angular velocity, and angular acceleration.

## 4. Method

### 4.1. Robot Operating System Footstep Planner

Developing an omnidirectional gait for bipedal robots is a highly challenging task due to several inherent complexities. These complexities include the high-dimensional dynamics of the robot, limitations in sensing and actuation capabilities, as well as computational constraints that require real-time processing. As a result, achieving an effective and efficient omnidirectional gait involves overcoming numerous technical hurdles. We propose a method of imitating target foot landing points to solve this challenging problem. Firstly, the paper describes the footstep state information using the global position and orientation of the bipedal robot’s supporting feet. Specifically, this information consists of a two-dimensional coordinate point and a heading vector θ provided by a sensor on the hip joint of one side. Next, the footstep planner in the ROS package is used to randomly generate a curve and export a set of 2D trajectory points (x,y,θ) with heading information, so that the bipedal robot’s foot landing points imitate those points, thereby achieving omnidirectional gait for the robot. To use the footstep planner to generate curve trajectories, a 2D grid map and initial and target poses (x,y,θ) are required as inputs. The paper sets the initial pose to the origin and generates 800 target foot landing points by randomly sampling from the target range (0,−1,−π/2) to (0,1,π/2) on an empty map.

### 4.2. Bipedal Periodic Objective Function

Bipedal gait typically exhibits a symmetrical character. During walking, leg movement can be segmented into swing and stance phases, with the stance phase further subcategorized into single-leg stance and double-leg stance periods. Thus, a gait cycle can be divided into two single-leg stance phases and two double-leg stance phases. To implement symmetrical gait in bipedal robot motion, the concept of periodic reward synthesis is employed. During the single-leg stance phase, one foot retains static contact with the ground while the other swings through the air. In the succeeding phase, the feet roles are interchanged, with the previously grounded foot now swinging and the previously swinging foot establishing supportive contact with the ground. As such, employing symmetry as a learning structure can improve learning efficiency. Symmetrical motion facilitates quicker convergence to more effective solutions and results in a more visually appealing bipedal gait. The policy objective needs to master walking ability in a simulated environment while also satisfying the requirement of gait periodic symmetry.

To manifest periodic locomotion for the bipedal robot and endow it with various gait styles, we designed a periodic symmetrical function based on expert experience. This function enables the robot’s left and right feet to track the lifted foot height outputted by the symmetrical function in real time. This function serves a dual purpose; it not only accomplishes periodic gait movement but also accelerates the convergence speed of the training process. Specifically, the mathematical expression of this function is shown in Equations (4) and (5).

(4) $$h_{t}^{r}=\{\begin{array}{c} 0,\;0<t<T_{std}\;or\;T_{h}<t<T\\ k_{s}h_{max}^{foot}{\left(\frac{t}{T_{s}}-k_{t}\right)}^{2}+\left(h_{foot}^{leg}-h_{max}^{foot}\right),\;T_{sd}\leq t\leq T_{h} \end{array},$$

(5) $$h_{t}^{l}=\{\begin{array}{c} 0,\;0<t<T_{std}+T_{h}\\ k_{s}h_{max}^{foot}{\left(\frac{t}{T_{s}}-k_{t}\right)}^{2}+\left(h_{foot}^{leg}-h_{max}^{foot}\right),\;T_{sd}+T_{h}\leq t, \end{array}.$$

where $h_{max}^{foot}$ represents the maximum height of the lifted foot, $h_{foot}^{leg}$ represents the height between the leg and ground at the initial pose, *T* is the period, $T_{h}$ is half of the period *T*, $T_{swing}$ is the period of the swing phase, and $T_{std}$ is the period of the stance phase. Moreover, the expression needs to satisfy $k_{s}*k_{t}^{2}=1$. We introduce a periodic symmetric objective $g_{t}$ to the policy. Finally, by mapping the given state $s_{t}$ and target $g_{t}$ to actions, the policy is modeled as $\pi (a_{t}|s_{t},g_{t})$.

### 4.3. Multi-Agent Systems and Policy Entropy

Reference [37] has shown that $p(s_{0})$ has a strong effect on the training outcomes of RL. An excellent distribution of $p(s_{0})$ can effectively reduce unnecessary exploration during the process of RL. Sampling the initial state from the data can enable the robot to execute turning maneuvers smoothly when an imitation robot simulates the walking data of the ROS footprint planner. Due to the differences in modeling between footstep data and robots, directly sampling $p(s_{0})$ from footstep data may not be appropriate for reproducing omnidirectional walking of imitation robots. The dependence on the quality of footstep data is significant for this trained strategy.

**Figure 2:**
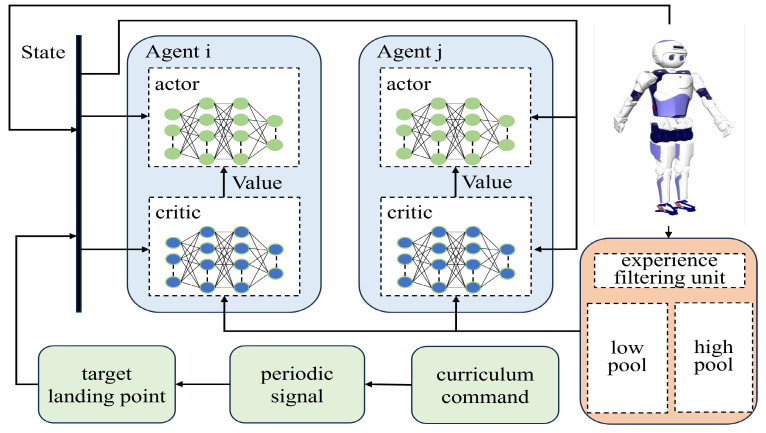
Training framework. Multi-agent collaboration solves the problem of initial state distribution differences. Policy entropy and experience replay mechanisms to improve training speed and quality. Course learning methods to implement multiple expressions of a single policy.

To mitigate the influence of data quality in ROS during the training phase, we propose learning the initial state distribution. This approach transforms the problem into a multi-agent RL problem, where all agents cooperate in fully cooperative tasks and share a common reward function. The training framework of our multi-agent RL method is illustrated in Figure 2.

The joint strategy of all agents is denoted as $\pi =\{\pi^{1},\pi^{2},\cdots ,\pi^{N}\}$. The primary objective of the cooperative task is for all agents to collaborate and find an optimal joint strategy $\pi^{*}$ that satisfies

(6) $$\pi^{*}=\arg\max_{\pi }\zeta \left(\pi \right),$$

where, $\zeta \left(\pi \right)=E_{\left(s_{t},a_{t}\right)∼\rho_{\pi }}\left[\sum_{t=0}^{+\infty }r\left(s_{t},a_{t}\right)\right]$ is the expected discount reward.

In a multi-agent environment, the state transition is jointly determined by the actions of all agents, and the reward obtained by each agent is interrelated with other agents. Therefore, altering the policy of a single agent directly impacts the selection of optimal decisions and the accuracy of value function estimation for other agents. To ensure convergence of the multi-agent system algorithm, we employ a centralized training-distributed execution architecture for training. Within this framework of centralized training and distributed decision making, we adopt an optimization approach that simultaneously maximizes cumulative reward and policy entropy to enhance exploration efficiency for each agent in an unknown environment. A policy entropy term can be added as

(7) $$\pi^{*}=\arg\max_{\pi }E_{\left(s_{t},a_{t}\right)∼\rho_{\pi }}\left[\sum_{t=0}^{\infty }r\left(s_{t},a_{t}\right)+\vartheta \varepsilon \left(\pi \left(\cdot |s_{t}\right)\right)\right],$$

where $\varepsilon \left(\pi \left(\cdot |s_{t}\right)\right)=-\log\left(\pi \left(a_{t}|s_{t}\right)\right)$; ϑ is the entropy coefficient.

The term “policy entropy” refers to the entropy of the policy distribution, which quantifies the level of randomness or exploration ability in an agent’s decision-making process. A higher policy entropy indicates a stronger inclination towards exploring unknown environments. This level of exploration is crucial to acquiring a comprehensive understanding of the environment and circumventing the risk of being trapped in suboptimal solutions. To strike a balance between exploration and exploitation, we introduce an optimization objective function for the entropy coefficient ϱ. The value of ϱ is updated using gradient descent, aiming to optimize the overall performance of the system. The objective function for optimizing the entropy coefficient is

(8) $$J\left(\vartheta \right)=E_{a_{t}∼\pi }\left[-\vartheta \log\pi \left(a_{t}|s_{t}\right)-\vartheta \varrho \right],$$

where ϱ is the action dimension of the agent.

The policy gradient of $p_{\omega }\left(s_{0}\right)$ is designed as

(9) $$J\left(\theta ,\omega \right)=E_{\tau ∼p_{\theta ,\omega }}\left[\nabla_{\omega }\log\left(p_{\omega }\right)\left(s_{0}\right)\sum_{t=0}^{T}\gamma^{t}r_{t}\right],$$

where $p_{\omega }\left(s_{0}\right)$ is the initial state distribution.

The motion control of the bipedal robot is governed by the actions of the first agent, which are determined by the policy $\pi (a_{t}|s_{t},g_{t})$. On the other hand, the second agent is responsible for proposing the initial state at the start of each round of RL training, as defined by the likelihood function $p_{\omega }\left(s_{0}\right)$. There is a cooperative relationship between the two agents. To maximize the multi-agent objective, the cooperative interaction between the two agents is expressed as

(10) $$J\left(\theta ,\omega \right)=E_{\tau ∼p_{\theta ,\omega }}\left[\Sigma_{t=0}^{T}\gamma^{t}r_{t}\right].$$

### 4.4. Heterogeneous Policy Experience Replay Mechanism Based on Euclidean Distance

Traditional experience replay mechanisms suffer from an imbalance in the distribution of experience data due to their reliance on a single experience buffer. This limitation often leads to prolonged training times required to achieve convergence. In many existing studies, the experience buffer is partitioned based on the quality of experiences, with a predominant focus on sampling from the pool of excellent experiences. However, this approach can lead to the model gradient overlooking the significance of ordinary experiences during the updating process, thereby narrowing the algorithm’s exploration range. Additionally, this division of experience may result in the disregard of valuable experience samples, thereby limiting the overall learning potential of the algorithm.

To address the aforementioned issues, we propose a novel heterogeneous policy experience replay mechanism based on Euclidean distance. This method tackles the problems by employing an experience filtering unit to store the generated experiences and conducting experience replay through two distinct experience pools: the low pool and the high pool. The former contains samples whose similarity is less than a preset threshold ϕ, while the latter contains those whose similarity is greater than ϕ. Initially, experience generated by training is stored in the experience filtering unit $F=(\varphi_{1},\varphi_{2},\cdots ,\varphi_{n})$, where $\varphi_{i}=\left(s_{i},a_{i},r_{i},s_{i+1}\right)$ consists of states and actions resulting from biped robot training. We establish an initial experience sample in *F* as a baseline experience sample. The similarity between the new experience sample and the baseline experience sample is then calculated using the Euclidean distance by:

(11) $$sim_{i}=\min(∥s_{i}-s_{o}{∥}_{2}+∥a_{i}-a_{o}{∥}_{2}).$$

We also design a sample similarity distance threshold ϕ to differentiate between similar samples, which is calculated as the average distance between new experience samples and baseline experience samples. In order to distinguish similar samples, a method is devised as:

(12) $$\phi =(sim_{1}+sim_{2}+\cdots +sim_{n})/n.$$

**Figure 3:**
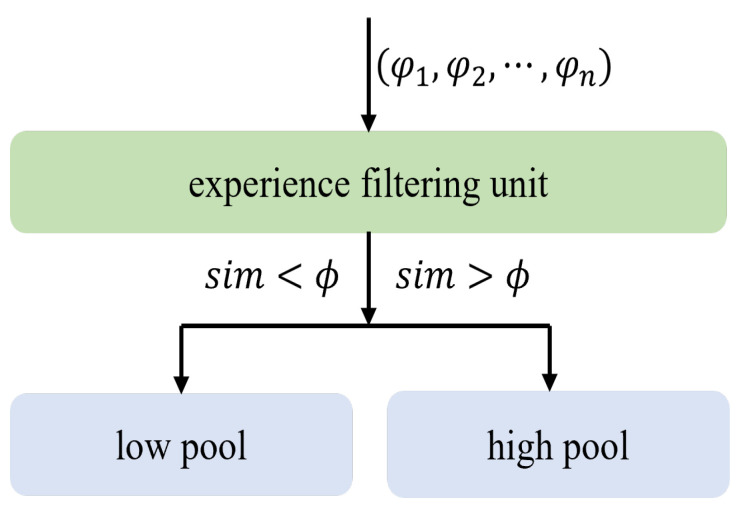
Heterogeneous policy experience replay mechanism. We establish an experience cache unit, baseline samples, and similarity thresholds.

The low pool stores experience samples with similarity less than ϕ, while the high pool is used to store experience samples with similarity greater than ϕ. In each round, experience samples are added to set *F*. At the end of each round, the experience filtering unit selects and categorizes the stored experience samples into their respective pools. Simultaneously, the benchmark experience samples are also updated. The sizes of the low pool and high pool are fixed. The probability of sampling and updating parameters from the high pool is α, while the probability from the low pool is 1−α. Compared to existing research, this heterogeneous strategy method not only maintains the role of the experience replay mechanism and expands the exploration range, but also enhances the influence of excellent samples on network gradients. Compared to [38], our dynamic baseline experience samples and similarity measure can to some extent filter out low-quality samples, thereby improving the model’s ability to learn from and generate high-quality samples. Additionally, the distribution of samples may change over time during the training stages of bipedal robots. The policy network may encounter different types of samples with varying features and importance. By continuously updating the baseline experience samples, we can better reflect the characteristics of samples in the current training stage, enabling the model to adapt to new data distributions and improve its generalization performance. Our experience replay mechanism is shown in Figure 3.

### 4.5. Design of Curriculum Learning Framework for Omnidirectional Gait

In this paper, we divide the difficulty of the task into various levels, which are determined by the magnitude of the turning angle. We propose an approach for selecting the appropriate difficulty level within the curriculum.

To generate new training tasks at the target difficulty level, we define the curriculum parameter set Λ as a sequence of gait training tasks arranged in ascending order of difficulty.

(13) $$\Lambda =\{l|0\leq l\leq l_{max}\},$$

where $l_{max}$ is the maximum level of difficulty in the curriculum.

In course strategy design, completion rate is used as a metric to gauge the current progress of the course. Additionally, it is also an important measure in evaluating whether one can advance to the next level of difficulty. We define the indicator function $S_{l}$, which uses course parameters to flag the task completion status.

(14) $$s_{l}=\{\begin{array}{c} 1,\;Task\;completed\\ 0,\;otherwise \end{array}.$$

The completion rate of a course under the course parameter *l* is defined as

(15) $$Pr\left(l\right)=\frac{\sum_{i=0}^{N}S_{l}\left(i\right)}{N}.$$

If a specific set of course parameter *l* satisfies Pr(l)>k, where *k* is a preset threshold for triggering, then the robot can complete the training task smoothly under this difficulty level. Afterwards, we elevate the task difficulty by increasing the angle of turning, and set the starting difficulty level for the next training session when resetting the environment.

With a course difficulty of *c* and a variance of $\sigma^{2}$, the density function of course *l* is

(16) $$\rho_{\sigma ,c}\left(l\right)=\exp(-\pi ∥l-c∥/\sigma^{2}),$$

where *c* determines the location of the distribution and $\sigma^{2}$ determines the magnitude of the distribution. Under l∈Λ, all $\rho_{\sigma ,c}$ discrete integrals are

(17) $$\rho_{\sigma ,c}\left(\Lambda \right)=\Sigma_{x\in \Lambda }\rho_{\sigma ,c}\left(l\right).$$

With a course difficulty of *c* and a variance of $\sigma^{2}$, the distribution function of course *l* is

(18) $$D_{\Lambda ,\sigma ,c}\left(l\right)=\frac{\rho_{\sigma ,c}\left(l\right)}{\rho_{\sigma ,c}\left(\Lambda \right)}.$$

Each thread selects an appropriate task difficulty level at each environment reset based on Equation (18). Our course learning steps are summarized in Algorithm 1. This method ensures that robots are not all put at the same difficulty level under each course difficulty level, but rather different environments with various difficulty levels are selected by discretizing according to a Gaussian distribution.
**Algorithm 1** Curriculum learning algorithm1:Initial curriculum difficulty2:The robot collects data and calculates whether the curriculum is completed or not by using Equation (13)3:Calculate the completion rate Pr(l) of the curriculum by using Equation (14)4:**If:**Pr(l)>k:5:   **If:** the highest difficulty level is reached:6:      Randomize the difficulty of the curriculum7:   **Else:**8:      Increase the difficulty of the curriculum9:**Else:**10:   Maintain the difficulty of the curriculum11:Return to step 2

### 4.6. Markov Decision Process Modeling for Omnidirectional Gait

(1) Statespace

The input to the controller in this paper is a 41-dimensional state space $s_{t}\in R^{41}$ including the pelvis orientation $\left(R_{roll},R_{pit},R_{yaw}\right)\in R^{3}$, the linear velocity $v_{p}=\left(v_{x},v_{y},v_{z}\right)\in R^{3}$ of the pelvis, the angular velocity $\omega_{p}$ of the pelvis, the target position of the pelvis $\left(p_{tarx},p_{tary},p_{tarz}\right)\in R^{3}$, the joint position $q\in R^{10}$ and joint velocity $\dot{q}\in R^{10}$ of each drive joint, and the target control velocity $v_{cmd}=\left(v_{x}^{cmd},v_{y}^{cmd},\omega_{z}^{cmd}\right)\in R^{3}$ of the robot. In addition, we include the next two target steps of the robot and its heading information $\left(x,y,\theta \right)\in R^{6}$. The phase vector is constructed as [sin((2∗pi∗t)/T),cos((2∗pi∗t)/T)].

(2) Action space

We use the PD control target as the action space and select the position of the movable joint as the action. Due to the underdrive of the robot, directly using PD control to apply the magnitude of the torque to track the reference angle will cause the robot to fall down easily. Therefore, we change the method of PD control and use position incremental control to solve the problem of robot underdrive. This method keeps the robot balanced by calculating the position increment of the robot and adjusting its torque. Using this method not only improves the stability of the robot, but also enables the robot to perform its tasks more flexibly and finely.

Therefore, our RL strategy outputs the incremental joint position $\sigma q_{t}$, which is added to the target joint position $q_{t-1}$ at the previous moment. Then the target joint position at the current moment is defined as

(19) $$q_{t}=q_{t-1}+\sigma q_{t}.$$

To track these joint angles, the torque applied to the joint is calculated using a low-level PD controller. The torque is calculated as

(20) $$\tau =k_{p}\left(q_{tar}-q\right)+k_{d}\left({\dot{q}}_{tar}-\dot{q}\right),$$

where $k_{p}$, $k_{d}$ are PD gains, which have been corrected in the actual simulation. $q_{tar}$ and $q_{tar-1}$ are the target position and target velocity of the joint, respectively. The linear velocity of the target joint is set to 0 during the training process. *q* and $\dot{q}$ are the current position and linear velocity of the active joint, respectively.

(3) Reward Functions

The purpose of the reward function is to motivate the robot to follow specified commands, keep its body smooth, and achieve stable motion. The reward function is designed as

(21) $$R=r_{vel}+r_{o}+r_{ad}+r_{\tau }+r_{alive}+r_{per}+r_{per}+r_{for},$$

Table 2 shows the details of the reward function of Formula 21. $h_{tar}^{foot}$ is the target height output by the cycle symmetric foot lift height curve imitated by the left and right feet; $h_{rel}^{foot}$ is the actual height of the left and right feet. $p_{tar}^{foot}$ is the target foothold; $p_{rel}^{foot}$ is the actual foothold. $p_{tar}$ is the target foothold; $p_{root}$ is the projection point of the actual position of the floating base. $r_{imi}$ encourages humanoid robots to place their feet on the target point. When the distance between one or both feet and the target point is within the target radius, it will be considered as stepping on the target point. $r_{for}$ keeps the distance between the projection of the root node and the target foothold constantly close, thereby preventing the robot from standing still, so that the floating base of the robot can continuously move to the next target point.

## 5. Results and Discussion

### 5.1. Experimental Environment and Settings

This experiment leverages the Ubuntu 20.04LTS operating system and the Pytorch deep learning framework. Our model is built upon the AMD Ryzen ThreadRipper 3990X CPU and the Nvidia GeForce RTX 3090 (24 GB) GPU. The MuJoCo platform serves as the foundation for our physical simulations, offering support for parallel learning, closed-chain or flexible robot simulation, and exceptional performance in various robot training tasks. During the experiment, we set the MuJoCo simulator to operate at a frequency of 500 Hz, the PD controller at 2000 Hz, and the neural network at 50 Hz. To expedite the training process, we simultaneously employed 16 parallel environments, allowing us to complete the training in approximately 6 h and collect a total of 40 million samples. Our novel approach incorporates curriculum learning, enabling the generation of different velocity commands with a single policy, without the need for policy retraining. Furthermore, we compared our method against both the direct adoption of the PPO method and the integration of traditional curriculum learning into the PPO method (CLPPO) in subsequent experiments. In order to guarantee consistent imitation behavior among the three sets of commands, we set the parameters *T*, $T_{std}$, and $T_{swi}$ to 30, 4, and 14, respectively. The $k_{p}$ and $k_{d}$ are set to 80 and 5, respectively. Our actor and critic networks both consist of two hidden layers, with 256 neurons in each layer. We parallelized the environment across 64 instances. The policy network is optimized every 512 steps, which corresponds to optimizing the policy network every 32,768 samples. We use a learning rate of 0.0003 and a discount factor of 0.98. Each round of training has 600 steps.

### 5.2. Straight Gait

**Figure 4:**
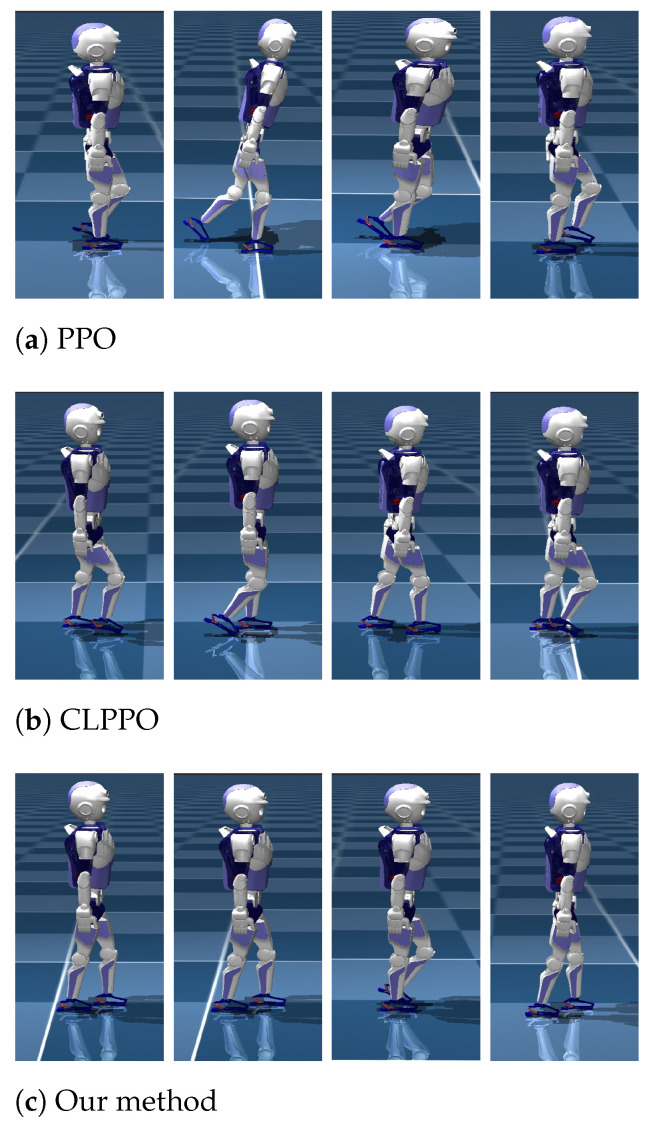
Walking state diagram of bipedal robot under three algorithms. We used the same straight-line speed command to conduct a comparison test of the robot’s walking using three algorithms.

**Figure 5:**
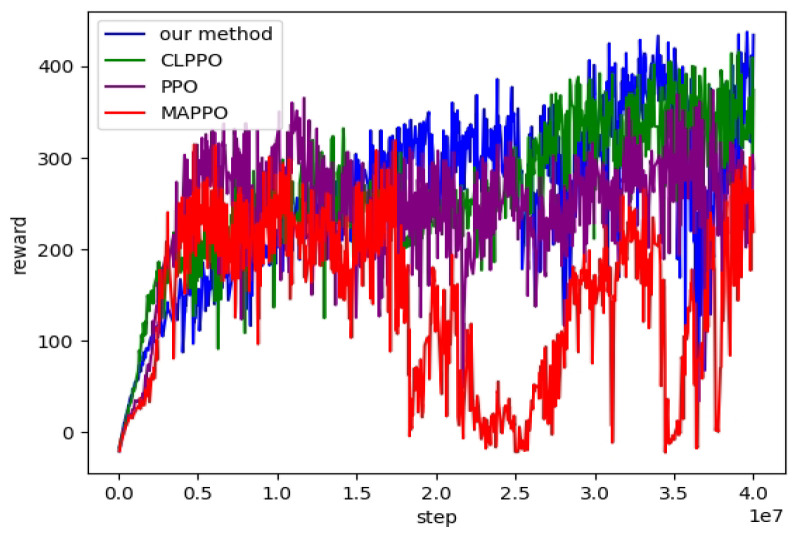
Training Process: A total of 40 million samples were collected to compare our algorithm with three other algorithms. The reward metric represents the average total reward value per iteration.

Figure 4 presents the state diagrams of the bipedal robot’s straight walking achieved by training its policy network with three different methods. In the performance comparison of the forward gait, the PPO method employs velocity commands $v_{cmd}=\left[0.5,0,0\right]$. Curriculum learning is incorporated into both CLPPO and our method based on these instructions. Both CLPPO and our method have a velocity command range of $v_{x}^{cmd}\in \left[-0.5,0.5\right]$, $v_{y}^{cmd}\in \left[-0.5,0.5\right]$, and $\omega_{z}^{cmd}\in \left[-0.5,0.5\right]$. It is evident from Figure 4 that the bipedal robot walking with the PPO method experiences noticeable sway. However, both our method and the CLPPO method do not demonstrate noticeable sway. We also compare the convergence of the three algorithms during the training process, as shown in Figure 5. We also include the training convergence of the multi-agent approach (MPPO) that treats the robot as an agent. The total reward of the MPPO method is the lowest among the four methods. Furthermore, only our method has a total reward exceeding 400. Therefore, only the two baseline algorithms PPO and CLPPO are compared below.

**Figure 6:**
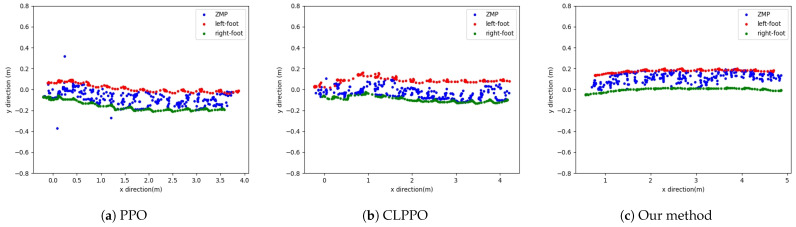
Diagrams of ZMP and positions of left and right feet. Testing robot straight motion using three algorithm-based training policies.

Figure 6 illustrates the ZMP position and the positions of the left and right feet for the three methods. Our proposed method maintains a consistent ZMP position between the touchdown points of the left and right feet, ensuring stability during bipedal robot locomotion. In contrast, the ZMP position under the PPO method displays significant jumps, while the CLPPO method effectively keeps the ZMP positioned between the two feet. The ZMP position under the PPO method is the least stable among the three methods, which is consistent with the significant body sway observed in Figure 4 due to the PPO policy control.

**Figure 7:**
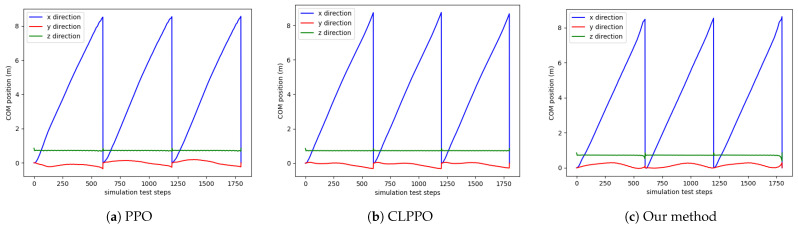
COM position diagram for the three methods. The position of COM in the three directions (x, y, z) during the walking process of the bipedal robot.

We conduct tests on the positions of the center of mass (COM) of the bipedal robot along the x, y, and z axes during the walking process. Each algorithm is reset after 600 steps during each execution, and a total of three rounds are tested, amounting to a total of 1800 steps. The results are presented in Figure 7. The change in the x-axis position under our method is smooth, indicating a consistent walking speed with no variations for the bipedal robot. Both the PPO and CLPPO methods show slight variations in the x-axis velocity, which result in body swaying during the robot’s walking process.

**Figure 8:**
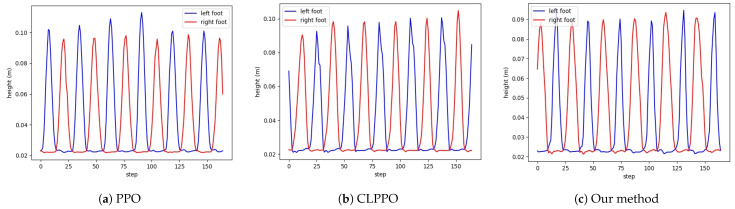
Height diagram of left and right feet for the three methods. Our method is basically the same height.

**Figure 9:**
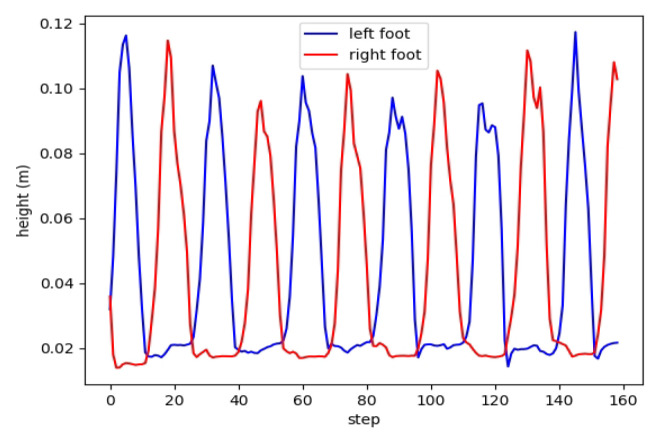
Height of left and right feet using the periodic gait method in reference [33]. The height of the foot lift is different.

From Figure 8a, it can be observed that PPO, which does not utilize the periodic reward designed by us, exhibits significant variations in foot height and fails to meet the periodic characteristics of bipedal walking. Our approach and CLPPO demonstrate consistent periodic variations in left foot height, as illustrated in Figure 8b,c. Failure to meet the periodic characteristics can lead to falls during the bipedal robot’s walking process. We also conduct tests using the cyclic gait method described in reference [33], as shown in Figure 9. However, due to the inability to specify foot lifting height, the periodic characteristics of foot elevation in bipedal robots cannot be satisfied.

### 5.3. Steering Gait

**Figure 10:**
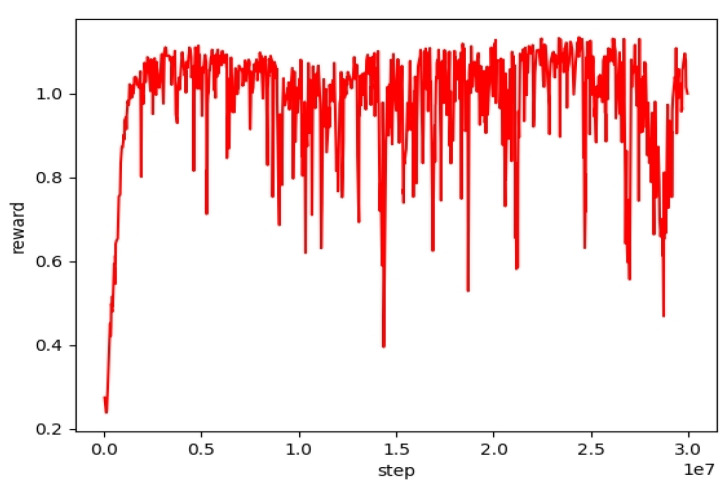
Training reward diagram of PPO method. Test our strategy by running in circles using $v_{cmd}=\left[0.5,0.1,0.1\right]$.

Next, we evaluate the steering gait performance of the three methods. We administer a steering velocity command $v_{cmd}=\left[0.5,0.1,0.1\right]$ to retrain the steering gait for the PPO method. CLPPO and our method continue to employ the previously trained policies. The PPO method displays convergence already in the early stages of training, as shown in Figure 10. Since the PPO method fails to achieve a steering gait for the bipedal robot, we exclusively compare the steering performance between CLPPO and our method.

**Figure 11:**
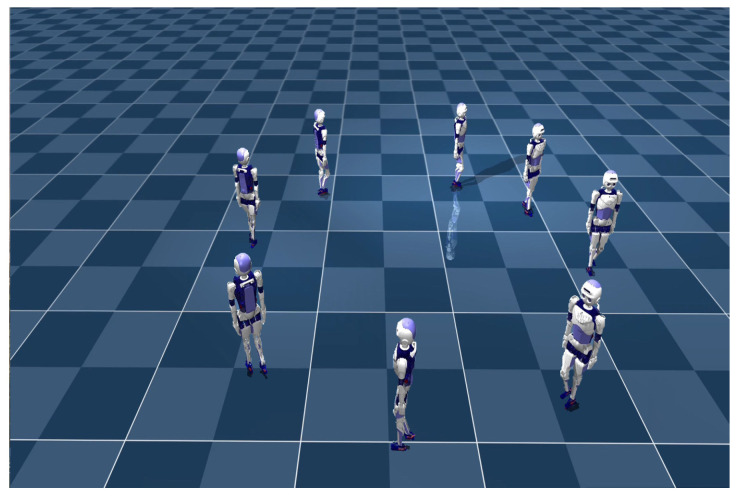
Walking state diagram of bipedal robot. The bipedal robot turns in circles at the same turning speed and finally returns to near the original place.

**Figure 12:**
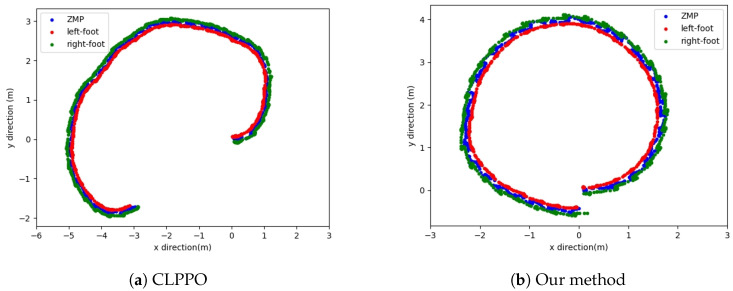
Diagrams of ZMP and positions of left and right feet.

The walking situation of our method in the simulation environment is shown in Figure 11. Figure 12 shows the ZMP positions of two methods and the positions of the left and right feet. Both methods can keep the ZMP position between the left and right feet, indicating that the robots can maintain stable walking. The oscillation in Figure 12a is caused by the inability of the robot to track the velocity command throughout the motion. This results in bipedal robots being unable to maintain smooth and continuous turning while walking, and the body of the robot also shakes when oscillating. However, our method controls the robot in turning and walking on a smoother trajectory without the oscillation observed in Figure 12b.

**Figure 13:**
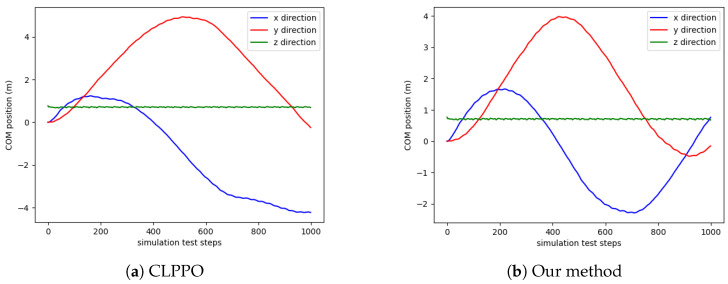
COM position diagram for the two methods.

Figure 13 shows the COM position of two methods. Our method ensures smoother variations in the robot’s position along the x-axis. This means that the robot can maintain a stable walking speed to ensure stability.

**Figure 14:**
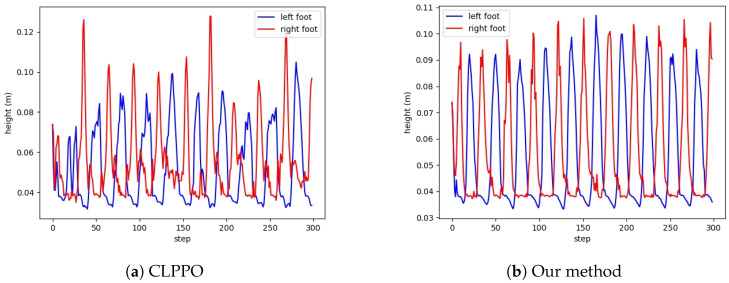
Height diagram of left and right feet for the two methods. Both methods executed the same speed command.

From Figure 14a, it is evident that there are pronounced discontinuities in the foot height of the bipedal robot when employing the CLPPO method. This does not guarantee the stability of the robot during the walking process. The foot heights during the steering process of our method are illustrated in Figure 14b. The foot heights of both feet continue to display periodic characteristics without experiencing substantial discontinuities, thus enabling a stable steering process.

**Figure 15:**
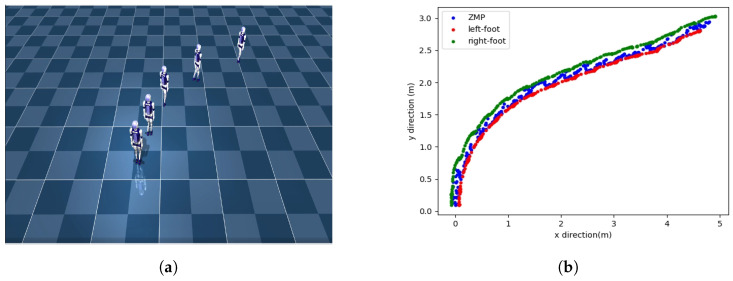
Robot walking gait switching diagram. The robot first goes straight, then turns, and finally continues going straight. (**a**) Scenario diagram of the robot in the simulation scene. (**b**) Diagrams of ZMP and positions of left and right feet.

**Figure 16:**
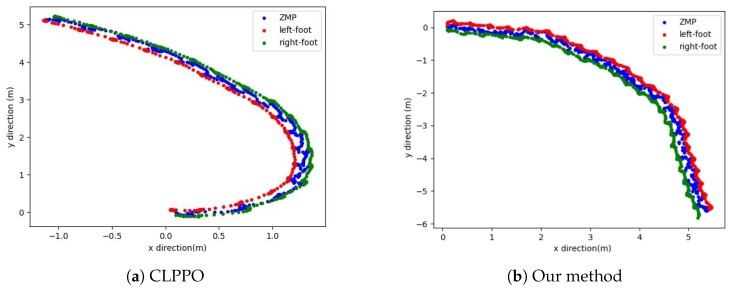
Our method performs gait switching tests between straight walking and turning in two steering modes.

Lastly, we test the performance of two methods in terms of omnidirectional gait. Firstly, we initiate straight walking; then, a rotational velocity command is given to the robot, transitioning it into straight walking after a certain duration. The CLPPO method experiences instances of backward movement and falling during the transition between the two gaits. In contrast, our method successfully executes the given velocity command independently, as shown in Figure 15a. The ZMP position and the positions of both feet in our method are illustrated in Figure 15b. Our method achieves smooth gait transitions through a single policy. We further validate the effectiveness of our method through additional velocity commands, as shown in Figure 16a,b. From the foot landing points and ZMP locations, it can be observed that our method ensures stability and smoothness during gait transitions.

## 6. Conclusions

In this work, we have proposed a novel RL framework for generating periodic symmetric bipedal omnidirectional gaits. The design of our bipedal periodic symmetric functions is crucial for achieving excellent gaits during the straight walking phase. Our approach leverages learning from the ROS footprint planner and curriculum learning techniques, enabling seamless transitions between omnidirectional gaits using a single policy network. The introduction of multi-agent RL mitigates the impact of differences between the footprint planner and our robot, allowing for parallel learning of initial state distributions for the agents. We have also incorporated policy entropy and heterogeneous experience replay mechanisms to expedite gait training for bipedal robots. The results from comparative experiments demonstrate that our method enables seamless transitions between different gaits for full-sized bipedal robots, not just smooth turning motions. Our approach achieves the smooth execution of omnidirectional gaits using a single policy, which is a significant accomplishment. Future research can explore more effective and robust methods for bipedal gait training. Robots need to walk on different terrains, such as stairs, slopes, and uneven surfaces. These terrains have different characteristics and difficulties, so it is necessary to design corresponding gait control strategies for different terrains. We will explore how to use techniques such as deep learning to automatically learn and optimize gait control strategies that adapt to different terrains. However, it is worth noting that although this study achieved omnidirectional gait of a bipedal robot in a reinforcement learning simulation environment, the ultimate goal of reinforcement learning is to simulate and transfer to the real world. We will try to solve this problem from two directions, of which one is to improve the robustness of the learned policies by changing the model parameters within a certain range in each iteration of the learning algorithm, and another is to combine learning with a model-based control.

## Figures and Tables

**Table 1:** The joint information of the robot.

Joint Name	Range of Motion	Peak Torque	Joint Velocity	Stiffness	Damping
Hip Yaw	−90${}^{\circ }$–90${}^{\circ }$	133 Nm	240${}^{\circ }$/s	0.1	1000
Hip Pitch	−60${}^{\circ }$–90${}^{\circ }$	45.9 Nm	576${}^{\circ }$/s	0.1	1000
Hip Roll	−10${}^{\circ }$–10${}^{\circ }$	5000 Nm	120${}^{\circ }$/s	0.1	1500
Knee Pitch	−140${}^{\circ }$–0${}^{\circ }$	48.6 Nm	540${}^{\circ }$/s	1	1500
Ankle Pitch	−80${}^{\circ }$–80${}^{\circ }$	28.8 Nm	918${}^{\circ }$/s	0.01	100

We only introduce information about the joints used in the lower body.

**Table 2:** Reward functions.

Effect	Expression
command tracking	$r_{vel}=exp\left(\left(v-v_{cmd}\right)/max\left(\delta \left(\Sigma {\left(v_{tar}\right)}^{2}\right),0.01\right)\right)$
keep balance	$r_{o}=exp\left(1-<q,\hat{q}{>}^{2}\right)$
smooth action	$r_{ad}=exp\left(\sum_{i=0}^{n}{\left(la_{i}-a_{i}\right)}^{2}\right)$
	$r_{\tau }=exp\left(\sum_{i=0}^{n}{\left(l\tau_{i}-\tau_{i}\right)}^{2}\right)$
Sport termination	$r_{alive}=\{\begin{array}{c} -1,\;ifstopconition\\ 0,\;\;\;\;else \end{array}$
Periodic Gait	$r_{per}=exp\left(\sum_{i=0}^{2}{\left(h_{tar}^{foot_{i}}-h_{rel}^{foot_{i}}\right)}^{2}\right)$
Omnidirectional gait	$r_{imi}=exp\left(\sum_{i=0}^{2}{\left(p_{tar}^{foot_{i}}-p_{rel}^{foot_{i}}\right)}^{2}\right)$
	$r_{for}=exp\left(-\sum_{i=0}^{2}{\left(p_{tar}-p_{root}\right)}^{2}\right)$

## Data Availability

The data that support the findings of this study are available from the corresponding author upon reasonable request.
